# Characterization of Genetic Determinants That Modulate *Candida albicans* Filamentation in the Presence of Bacteria

**DOI:** 10.1371/journal.pone.0071939

**Published:** 2013-08-07

**Authors:** Sean J. Fox, Bryce T. Shelton, Michael D. Kruppa

**Affiliations:** Department of Biomedical Sciences, Quillen College of Medicine, East Tennessee State University, Johnson City, Tennessee, United States of America; University of Aberdeen, United Kingdom

## Abstract

In the human body, fungi and bacteria share many niches where the close contact of these organisms maintains a balance among the microbial population. However, when this microbial balance is disrupted, as with antibiotic treatment, other bacteria or fungi can grow uninhibited. *C. albicans* is the most common opportunistic fungal pathogen affecting humans and can uniquely control its morphogenesis between yeast, pseudohyphal, and hyphal forms. Numerous studies have shown that *C. albicans* interactions with bacteria can impact its ability to undergo morphogenesis; however, the genetics that govern this morphological control via these bacterial interactions are still relatively unknown. To aid in the understanding of the cross-kingdom interactions of *C. albicans* with bacteria and the impact on morphology we utilized a haploinsufficiency based *C. albicans* mutant screen to test for the ability of *C. albicans* to produce hyphae in the presence of three bacterial species (*Escherichia coli*, *Pseudomonas aeruginosa*, and *Staphylococcus aureus*). Of the 18,144 mutant strains tested, 295 mutants produced hyphae in the presence of all three bacterial species. The 295 mutants identified 132 points of insertion, which included identified/predicted genes, major repeat sequences, and a number of non-coding/unannotated transcripts. One gene, *CDR4*, displayed increased expression when co-cultured with *S. aureus*, but not *E. coli* or *P. aeruginosa*. Our data demonstrates the ability to use a large scale library screen to identify genes involved in *Candida* -bacterial interactions and provides the foundation for comprehending the genetic pathways relating to bacterial control of *C. albicans* morphogenesis.

## Introduction

Polymicrobial communities of bacteria and fungi play a pivotal role in both human health and disease and can be found on nearly all facets of the human body including the skin, mouth, lungs, gastrointestinal, urinary, and reproductive tracts [1]. As varied as the habitats these microbes reside in are the types of interactions (both chemical and physical) that the opportunistic fungal pathogen *Candida albicans* and bacteria have with one another, which are only recently being fully appreciated by the scientific community [1,2]. *C. albicans* ability to change morphology from a budding yeast to a filamentous hyphae is considered a major virulence factor [3,4] that is influenced through a variety of environmental and host factors, including temperature, serum, pH, nutrient availability, and quorum sensing (QS) [5]. QS in *C. albicans* is regulated by the molecule farnesol [6], which when present at saturating levels, inhibits the ability of *C. albicans* to shift from yeast to hyphal form. This type of chemical communication among a population has also been well documented in bacteria [7–10]. Recent research has shown that bacterially secreted quorum sensing molecules (QSMs) and other metabolites can influence *C. albicans* morphology as well. For example, *Pseudomonas aeruginosa* secretes the QS molecule (QSM) 3-oxo-C12 homoserine lactone (3OC12HSL) that can block *C. albicans* filamentation [11]. Likewise, *Burkholderia cenocepacia*’s diffusible signal molecule, *cis*-2 dodecenoic acid, has been shown to inhibit *C. albicans* germ tube formation [12]. Similar inhibitory effects can be observed when *C. albicans* interacts with the bacteria *Acinetobacter* *baumannii* and *Streptococcus mutans* or the yeast *Saccharomyces* *boulardii* [13–15]. Interestingly, different bacterial species from the same genus, as in the case of *Streptococcus* , can have opposing effects on *C. albicans* morphology. For instance, the oral bacterium *S. mutans* inhibits hyphal formation of *C. albicans* via the QSMs trans-2-decenoic acid and competence stimulating peptides [14,16]; however, *S. gordonii* appears to stimulate *C. albicans* hyphae formation in an effort to aid in the colonization of the oral cavity [17]. These results suggest that *C. albicans* has mechanisms in place that can recognize these bacterial molecules in the environment and respond accordingly.

Physical interactions between bacteria and *C. albicans* include both attachment and the development of mixed species biofilms on both biotic and abiotic surfaces [18]. In addition to *P. aeruginosa* secretion of 3OC12HSL, the bacterium preferentially attaches to and kills the *C. albicans* hyphal form, but not the yeast form [19]. Alternatively, *Staphylococcus epidermidis* enjoys the ability of attaching to both the yeast and hyphal forms of *C. albicans*, and when found together as in a mixed biofilm, enhances *C. albicans* resistance to fluconazole [20]. Additionally, *S. aureus* preferentially attaches to *C. albicans* hyphae and these mixed biofilms show increased drug resistance to Vancomycin [21–23]. On the other hand, farnesol has been shown to disrupt *S. aureus* membrane integrity making it more susceptible to antibiotics as well as interfering with biofilm growth [24]. From a virulence standpoint, co-infection of *C. albicans* and other bacterial species, including *S. aureus, E. coli* and *P. aeruginosa*, show synergy with increased mouse mortality when the organisms are infected at sublethal doses [23,25–28]. Furthermore, prior colonization of *E. coli* in the urinary tract enhances *C. albicans* ability to colonize and subsequently cause urinary tract infections where typically it is unable to do so [29]. These trans-kingdom interactions may indicate that microorganisms utilize the nearby molecules to sense and monitor their shared surrounding, adapt to changes in the local environment, and survive within a mixed species population. Despite the recent influx of research into *Candida* -bacterial interactions little is known about the genetics behind the mechanisms of communication that govern these interactions, and how they control morphological change in *C. albicans*.

Multiple species of bacteria are known to inhibit *C. albicans* filamentation and our goal was to identify mutants that did not respond when cultured in the presence of bacteria. In the present study, we utilized a large scale haploinsufficiency based screen to identify the genetic elements regulating *C. albicans* filamentation in the presence of bacteria. To our knowledge, this screen is the first to identify genetic determinants involved in polymicrobial interactions of *C. albicans* with bacteria and how they control morphogenesis of *C. albicans*. We identified 132 different genetic elements that appear to be involved in the ability of *C. albicans* to filament in the presence of three different bacteria (*E. coli, P. aeruginosa*, *S.* aureus). The results from this screen begin to offer a better understanding of the genetics behind *Candida* -bacterial interactions as well as factors influencing the morphogenesis of *C. albicans*.

## Materials and Methods

### Strains, media, and growth condition

*C. albicans* wild type SC5314 [30], was routinely cultured on Yeast Peptone Dextrose (YPD) medium (2% dextrose, 2% peptone, 1% yeast extract, 2% bacto agar) at 30° C. The *CDR4* deletion strains [SFLUC6(Δ*cdr4/CDR4*) and SFLUC4(Δ*cdr4/Δcdr4*] have been previously described by Morschhäuser et al. [31]. The *ALS6* deletion strains [1522(Δ*als6/ALS6*) and 1420 (Δ*als6/Δals6*)] have been previously described by Hoyer et al. [32]. *E. coli* (ATCC#33922), *P. aeruginosa* (ATCC#27853), and *S. aureus* (ATCC#25923) strains were maintained on Luria Broth (LB) medium (1% tryptone, 1% NaCl, 5% yeast extract, 2% bacto agar) at 37° C.

### Screen for filamentation mutants

A transposon insertion library of 18,144 *C. albicans* strains was constructed using *C. albicans* strain CAI4 [33] and a Tn7 plasmid insertion library created by Uhl et al [34]. To perform the screen for filamentation, the library strains were individually spot replicated, with two centimeter spacing, onto YPD, Medium 199 (M199) (9.5g medium 199 with Earles salts, 18.7g Tris-HCl, 20g bacto agar, pH 7.5), or M199 plates that had been pre-coated with bacterial lawns from fresh 37° C overnight cultures. The YPD plate was incubated at 30° C for 48 hours and served as a positive control for growth and a negative control for filamentation. The M199 control and bacterial plates were incubated at 37° C and were monitored for filamentation for up to seven days. The M199 control plate (without bacteria) served as a positive control for filamentation. Library candidates that filamented in the presence of bacteria were compared to the wild type control (SC5314) and retested twice under the same conditions to confirm the filamentous phenotype.

### Filamentation in liquid media

*C. albicans* strains were grown overnight at 30° C, washed three times with dH_2_O and cells counted with a hemocytometer. 1x10^6^ cells/mL were inoculated into pre-warmed medium 199 7.5 (37° C, control), or medium 199, pH7.5 containing bacteria that were pre-grown overnight at 37° C. This provided a high concentration of bacteria to simulate crowding conditions for *C. albicans*. For spent media the bacteria were spun out by centrifugation 5 min at 8,000 x *g*, then the medium was filtered to ensure removal of any remaining bacterial cells. The spent media was then inoculated with the *C. albicans* strains. The *Candida* *strains* were incubated for 2.5 hr at 37° C and morphology was assessed microscopically.

### Mapping of transposon insertion sites

*C. albicans* library candidates that filamented in the presence of all three bacteria were inoculated into 5mL of YPD and incubated overnight at 30° C with shaking (155 rpm). Cells were harvested by centrifugation and chromosomal DNA was obtained using a standard bead extraction [35]. Chromosomal DNA was digested with *Bsrg*I (New England), diluted 1:100, and treated with T4 DNA ligase (New England) to allow for the reconstitution of the original insertion plasmid. The ligated DNA was then transformed into *E. coli* TB-1 cells that were made competent with calcium chloride [36]. The transformed cells were plated onto LB agar plates with 50 µg/mL ampicillin and incubated overnight at 37° C. The successfully transformed cells were then grown overnight in 5 mL of LB with 50 µg/mL ampicillin and plasmid DNA was purified using the Promega Wizard^©^ Plus DNA purification system. Purified plasmids were sequenced using primers MKOL544: 5’-GATCTGAGTGAGCATCAACAG-3’ or MKOL525 5’- GCTATGACCATGATTACGCCAGG-3’ that recognize the 5’ and 3’ flanking sequences of the transposon allowing for sequencing on either side of the insertion point. The resulting DNA sequences were used to search the *Candida* Genome Database by BLAST comparison to identify the region of transposon insertion [37].

### Reverse transcription and gene expression

*C. albicans* SC5314, *E. coli*, *P. aeruginosa*, and *S. aureus* strains were grown in 50 mLs of YPD at either 30° C or 37° C until they reached mid-log phase. Cells were then harvested by centrifugation, washed with 1X PBS, and resuspended in 50 mLs of M199. *C. albicans* SC5314 and single species bacteria were then combined in equal amounts and incubated (at 30° C or 37° C) with shaking (155rpms). Aliquots were taken at 0, 10, 20, 30, 60 minutes post addition, cells were harvested, and samples were frozen. Acid phenol RNA extraction was performed on the samples to extract total RNA [35]. Reverse transcription PCR was performed using a Verso 1-Step RT-PCR kit (Thermo-Fisher). Primers MKOL597 5’-GGCAGATGCC GATACGAGTTCAAATTCG-3’ and MKOL598 5’- CATCAGAAGCCGAACC ATAAGCACGC-3’ were used for *CDR4* RNA detection. The *ENO1* gene served as a loading control using primers MKOL188 5’ –CGACTCCAGAGGTAACCC- 3’ and MKOL189 5’ – CCCAAGCATCCCAGTC -3’. Primers for ALS6 detection were used as described by Zhao et al [38]. Images were captured using a Syngene G: Box system and analysis was performed using Gene Tools software by SynGene. The experiments were performed three times, the data were quantified and statistically analyzed by a student’s t-test. P-values were calculated and those that were significant (P<0.05) were noted.

## Results

### Identification of 295 *C. albicans* mutant strains that filament in the presence of three bacterial species

**Figure 1:**
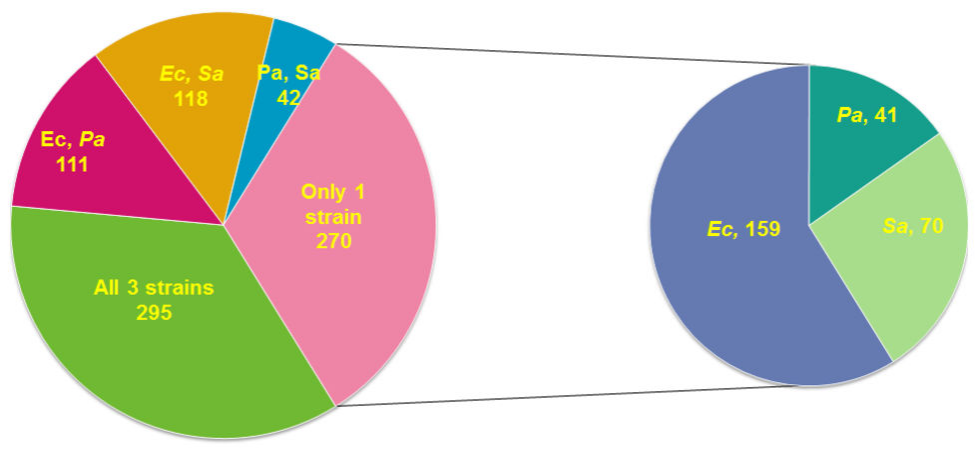
Distribution of mutants that filament in the presence of the three bacterial species tested. Overall 836 mutants were identified that filamented in the presence of the three bacterial species tested. Ec:*E*. *coli*; Pa: *P. aeruginosa*; Sa: *S. aureus*.

**Figure 2:**
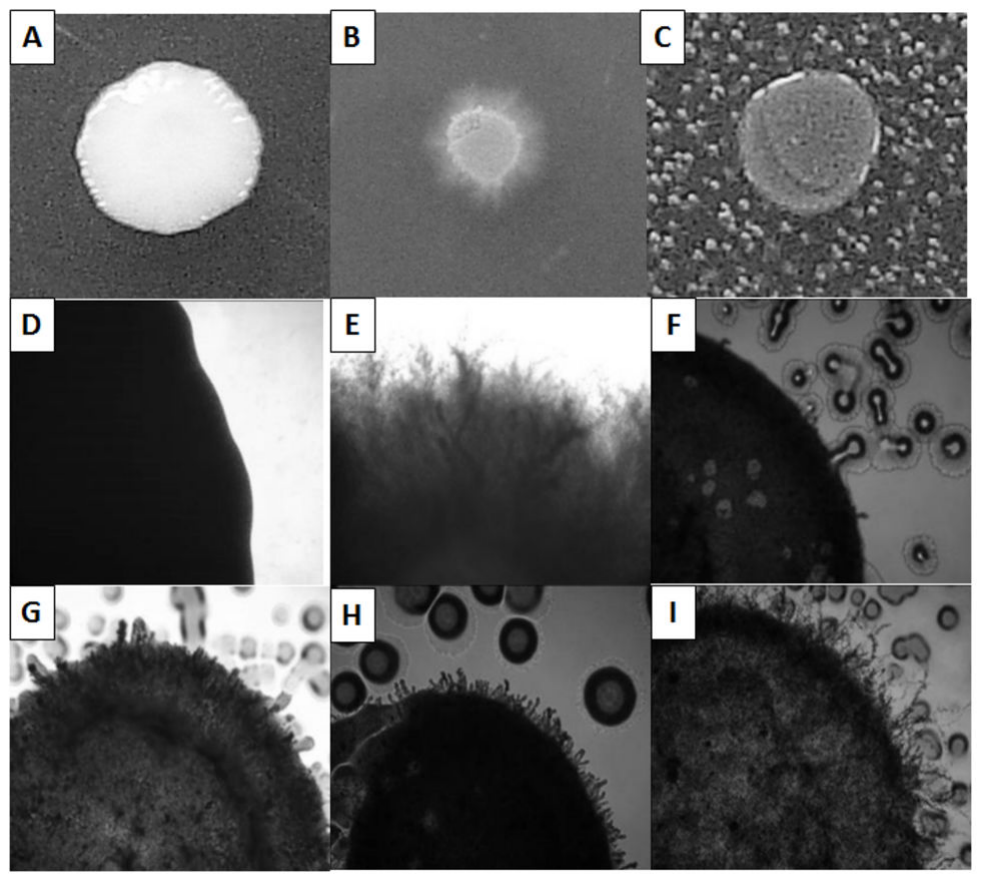
Representative colony morphologies. (**A**) SC5314 wild-type non-filamentous growth on YPD agar (30° C); (**B**) SC5314 wild-type filamentation on M199 agar (37° C); (**C**) SC5314 wild-type filamentation inhibition in the presence of *P. aeruginosa* on M199 agar (37° C); (**D**) 4X magnification of A; (**E**) 4X magnification of B; (**F**) 4X magnification of C; (**G**), (**H**), (**I**) Representative examples of library mutant filamentation in the presence of bacteria due to haploinsufficiency on M199 agar (37° C) (4X magnification).

To identify the genetic elements involved in *C. albicans* polymicrobial interactions, we utilized a *C. albicans* transposon insertion mutant library previously described by Uhl et al [34]. The library was spot replicated, along with a wild-type (SC5314) control onto agar plates of YPD, M199, and M199 containing a lawn of either *E. coli*, *P. aeruginosa* or *S. aureus*. To control for hyperfilamentous mutants and remove them from the screen, strains were grown on YPD at 30° C while mutants that did not filament were screened out on M199 at 37° C. We chose the test conditions of growing the strains on freshly spread bacterial lawns as this would result in a competitive growth condition for the *Candida* strains in the presence of bacteria. Our choice of the Gram positive, *S. aureus*, and the two Gram negative, *E* *. coil* and *P. aeruginosa*, was due to their association with host environments that *C. albicans* is often known to colonize. We scored the strains to determine if the *C. albicans* mutants filamented in the presence of one, two or all three bacterial species. Overall we identified 836 strains (4.60% of the entire mutant population) that filamented in the presence of one or more of the bacteria (Figure **1**). The systematic screening of the transposon insertion library identified 295 individual strains that produced a filamentous phenotype in the presence of all three bacterial species. We also identified 271 strains that filamented in the presence of two of the three bacterial species and 270 that only filamented in the presence of one species of bacteria. We decided to focus our efforts on the mutants that filamented in the presence of all three bacterial species as these mutants would likely be linked to a common mechanism for *C. albicans* to respond to different species of bacteria. Figure **2** depicts representative phenotypes of both the wild-type control as well as a *C. albicans* transposon library strains that produced filaments in the presence of bacteria. On YPD at 30° C, the SC5314 control shows a colony’s typical yeast, non-filamentous, morphology with characteristic smooth rounded edges (Figure **2 A, D**) while its growth on M199 without bacteria at 37° C shows hyphal filaments protruding from the colony (Figure **2 B, E**). SC5314 grown in the presence of high concentrations of bacteria lack filaments around the colony (Figure **2 C, F**) while the mutants identified by our screen exhibit filamentation in the presence of all three bacterial species (Figure **2 G–I**).

### Localization of the Tn7 transposon insertions

To identify the region of genome that the transposon insertion was located, the recovered insertion plasmid constructs were sequenced and the resulting sequences were then mapped by BLAST search of the Candida Genome Database (CGD) [37]. The 295 library candidates were mapped to 132 points of insertion within genes and other genetic components of the *Candida* genome, multiple independent candidates were shown to map to the same gene further validating the genetic screen. Fifty percent of the Tn7 insertions mapped within an open reading frame (ORF) while 18% of the insertions mapped to the 5’ upstream region and 13% to the 3’ downstream of an ORF (Table **S1**). This accounted for 107 known/predicted genes that fell into an array of categories related to gene function including enzymatic activity, transport, transcription, signaling, and adhesion (Table **1**). By far, the majority of identified genes and ORFs fell into the category of unknown function. From our screen we noted that the majority of the candidates we identified had not been previously associated with filamentation in general. However the *SSU1*, *FGR10*, *FGR24*, *RAS2*, *MRP2*, and *CCR4* haploinsufficient mutants we identified in our screen also overlap with the dataset from a haploinsufficient library screen performed by Uhl et al. [34] for filamentous mutants. In their study, Uhl et al [34] screened for hyperfilamentous and less filamentous mutants using different conditions than we utilized in this study. These mutants did not show any hyperfilamentous or less filamentous phenotypes under our control conditions indicating variability between the two experimental approaches.

**Table 1:** Biological process categories of genes identified from library screen.

**Biological Process**	**Gene/orf identified**
Enzymatic activity	*ALK8, FMO1, GCA2, GOR1, GCV1, IAH1, PLC1, orf19.346, orf19.511, orf19.1110*,
	*orf19.2114, orf19.4112, orf19.4246, orf19.5169, orf19.5665, orf19.7152*
	*orf19.7512*
Adhesion	*ALS6, EAP1, PGA28, orf19.5813*
Cell wall associated	*BMT8, GSC1, GSL1, PGA52*
Cell cycle	*IRR1, MAD2, NUF2, TEM1*
Signal transduction	*CAS4, RAS2, TOR1*
Transport	*CDR4, CRM1, GNP3, MGE1, POM152, RTA3, SSU1, orf19.6592, orf19.6747*
Transcription	*BDF1, CCR4, CTA24, CRZ2, HAP31, PHO23, SPT7, SUA72, ZCF11, orf19.536*
	*orf19.470*
Translation	*MRP2, TIF5, orf19.4176*
Protein processing	*DNM1, orf19.3730, orf19.3767, orf19.4086, orf19.5212, orf19.6082*,
	*orf19.7358*
Proteolysis	*AXL1, FGR10, orf19.4610*
RNA processing	*ILS1, NHP2, POP3, SEN1, orf19.1201, orf19.6736, orf19.6931*
RNA binding	*orf19.3124, orf19.4018*
DNA binding	*orf19.2579, orf19.7301*
DNA repair	*MEC3, SMC5, orf19.4412, orf19.6722, MMS21*
Ribosomal associated	*SOF1,RPS12, RPS18*
Amino acid synthesis	*CHA1, ECM17, HPA2*
Autophagy	*SPO72, orf19.2982*
Unknown	*FGR34,orf19.344, orf19.1368, orf19.1728, orf19.2038, orf19.2106, orf19.3087.2,*
	*orf19.3100, orf19.3394, orf19.3643, orf19.4263, orf19.5799, orf19.5897*
	*orf19.6488, orf19.6968, orf19.7085, orf19.7130, orf19.7567*

A number of insertions (5%) were mapped to the RPS and RB2 repeat regions located within the major repeat sequences (MRS) of MRS-1, -2,-4,-6, -7a, -7b, and –R units (Table **S2**). There were no Tn7 insertions found within MRS-5, nor the partial RB-2-4a repeat unit. Within the RB2 repeat region, Uhl et al previously identified a family of genes, *FGR6*, which have no clearly defined function other than their association with fungal growth [34]. It appears that at least seven of eight *FGR6* family members (*FGR6-1*, *FGR6-3*, *FGR6-4*, *FGR6-10*, orf19.727, orf19.6896, orf19.5775) are involved in regulating filamentous growth in the presence of bacteria. Finally, 14% of the insertions were mapped to non-coding transcripts or unannotated ORFs (Table **S3**). Many of these transcripts were characterized previously by RNA-seq analysis [39] and tiling arrays [40], but have not been further characterized regarding their functional roles in the cell. We use the term “transcripts” at this time as the transcribed regions have not been designated officially as protein coding ORFs or regulatory RNA genes in the CGD.

### Confirmation of phenotypes for Δ*cdr4/CDR4* and Δ*als6/ALS6* haploinsufficient mutants

**Figure 3:**
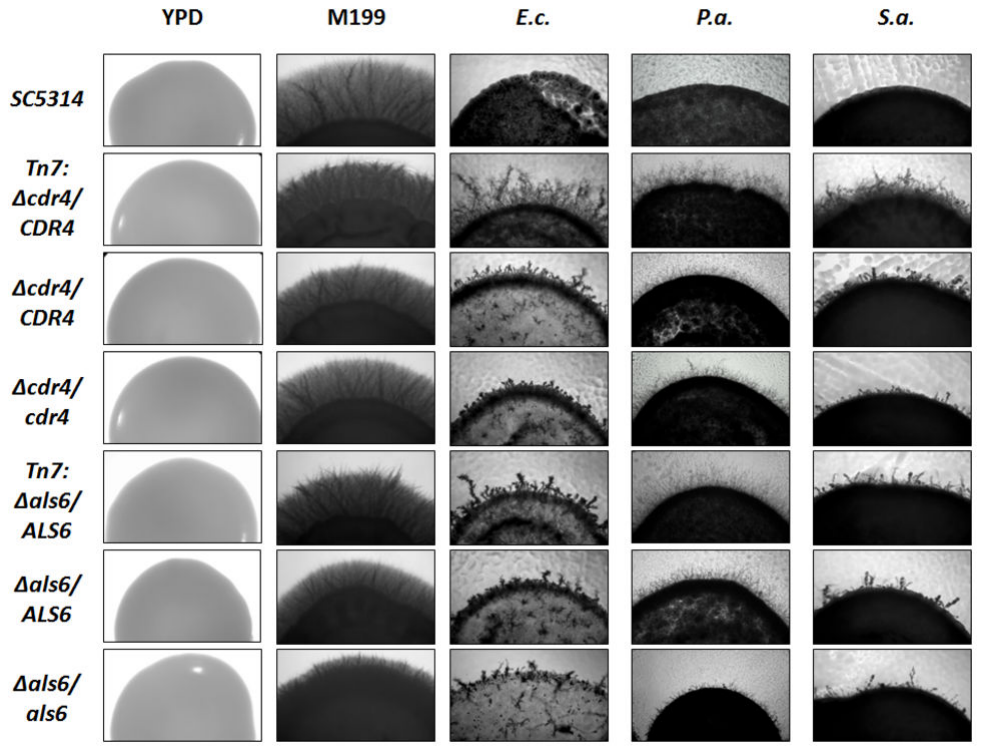
Representative photos of observed phenotypes of SC5314, library transposon candidates, and heterozygous and homozygous deletion strains. YPD growth control (30° C), 40x magnification; M199 filamentation control (37° C), 100x magnification; *E. coli* interactions with *C. albicans* strains, 100x magnification; *P. aeruginosa* interactions with *C. albicans* strains, 100x magnification; *S. aureus* interactions with *C. albicans* strains, 100x magnification.

**Figure 4:**
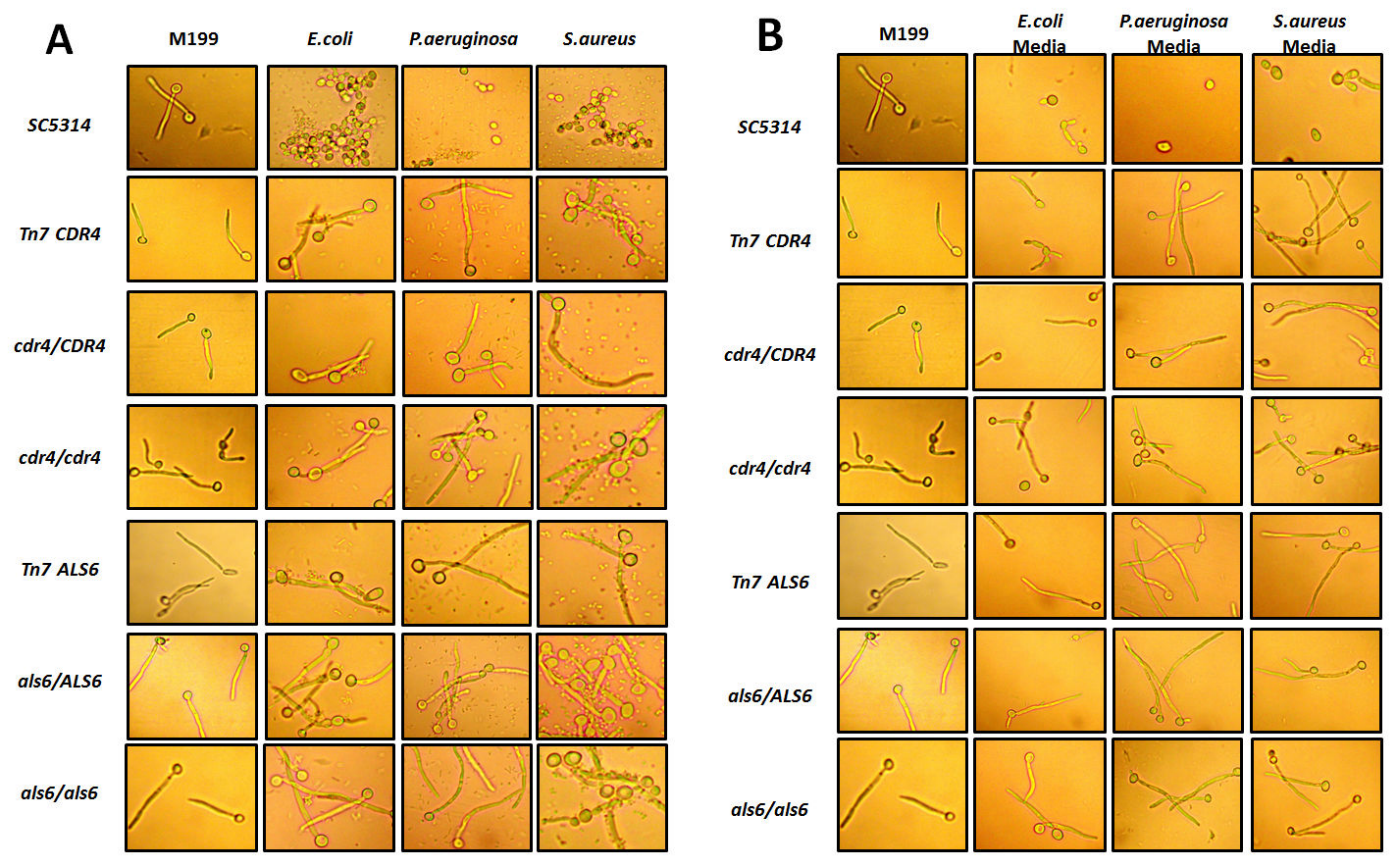
Phenotypes observed when SC5314, library transposon candidates, heterozygous and homozygous deletion strains grown in liquid culture (37° C) with bacteria or spent media. A) *C. albicans* strains with M199 filamentation control and coculture with *E. coli*, *P. aeruginosa*, and *S. aureus*. Magnification 400x; B) *C. albicans* strains with M199 filamentation control and culture in spent media *E. coli*, *P. aeruginosa*, and *S. aureus*. Magnification 400x.

Although we identified independent insertions near or within the same gene multiple times from this screen, to further confirm our observed phenotypes we chose two mutants*, Δals6/ALS6* and *Δcdr4/CDR4*, to be tested using independently constructed heterozygous and homozygous null strains. The *ALS6* mutant was chosen as the original Tn7 library insertion point was in the promoter region of this gene, while the *CDR4* mutant was chosen as the insertion fell within the open reading frame. The *als6* and *cdr4* heterozygous and homozygous null strains (obtained from Drs. Hoyer and Morschhäuser) were spot replicated with our haploinsufficient *Δals6/ALS6* and *Δcdr4/CDR4* library strains onto YPD, M199, and M199/bacterial plates using the same conditions from the original library screen. The phenotypes of the *als6* and *cdr4* heterozygous and homozygous null strains were similar if not identical to those we saw with our Tn7 mutant isolates (Figure **3**), thereby confirming our original observations and the validity of using haploinsufficiency for characterizing bacterial- *Candida* interactions. Furthermore, we examined the morphology of the strains in liquid culture in the presence of bacteria and spent media (Figure **4**). When cells were cultivated under hyphal inducing conditions in M199 we observed all strains exhibiting a hyphal morphology, whereas the control strain showed clear impairment of filamentation when grown in the presence of all three bacterial species. All the mutant strains filamented in the presence of all three bacterial species with hyphae being the predominant morphology (Figure **4A**). Also when *C. albicans* was inoculated into spent media that the bacteria had been removed from the mutants were capable of filamenting as hyphae and the wild type strain was impaired as mainly yeast and some pseudohyphae (Figure **4B**). This result also suggests that primary mediator(s) inhibiting *C. albicans* filamentation are secreted actively by the bacteria.

### *C. albicans* CDR4 expression increases in the presence of *S. aureus*

**Figure 5:**
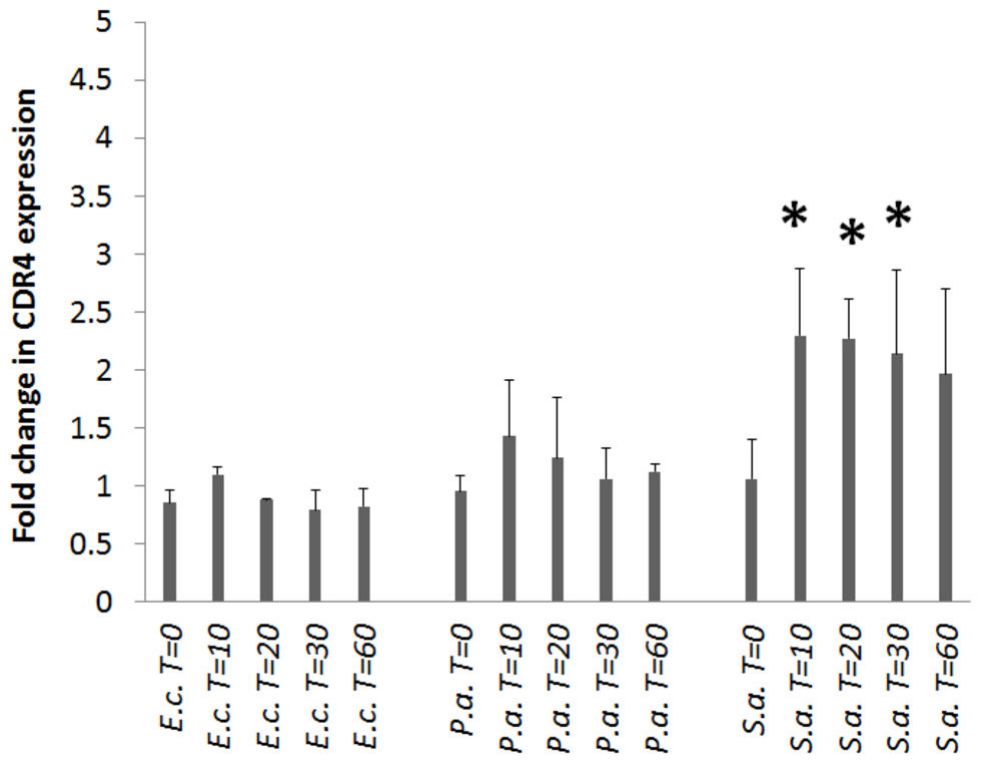
*C. albicans CDR4* transcript levels grown in the presence of bacteria. *C. albicans* SC5314, *E. coli*, *P. aeruginosa*, and *S. aureus* were grown to mid log phase in liquid culture, mixed together in equal amounts, co-incubated at 37° C, and aliquots were taken at 0, 10, 20, 30, and 60 minutes post addition. RNA was isolated and expression of *CDR4* was measured by reverse transcription. *ENO1* was used as a loading control and reference gene for expression comparisons. Graphical representation of *CDR4* expression over time for SC5314 co-incubated with *E. coli*, *P. aeruginosa*, or *S. aureus*. Data is representative of three independent experiments with mean value and standard deviation bars shown. The asterisks indicate a statistically significant difference (P<0.05) in mean intensity of test conditions over the control.

*C. albicans* and the individual bacterial strains were co-cultured and *CDR4* transcript levels were analyzed over time. The SC5314 cells co-incubated with *S. aureus* at 37° C exhibited over a two-fold increase in *CDR4* expression over the time course of 60 minutes as compared to the SC5314 control without bacteria (Figure **5**). There was little to no change in *CDR4* expression in the presence of *E. coli* or *P. aeruginosa*, nor was there any change in *CDR4* expression when tested at 30° C with any of the three bacterial species (data not shown). We also observed that spent bacterial culture media (bacteria removed by filtration) was unable to stimulate the expression of *CDR4* (data not shown). This suggests that a combination of physiologic temperature and (in the case of *S. aureus*) some sort of contact between the organisms is required for *CDR4* induction. Others have shown that changes in morphology and growth may require live bacteria to be present in the media to have a full inhibitory effect on *Candida* filamentation [13]. This would suggest that *C. albicans* interactions with bacteria are multifactorial encompassing both contact dependent mechanisms as well as secreted molecular factors. We also examined the expression of *ALS6*, however no increase in transcript level was observed with any of the growth conditions (data not shown).

## Discussion

The microbiome of the human body plays an important role in our health. Currently, studies involving the microbiota have only examined what the microbial population structures appear to be in both healthy and diseased individuals [41–43]. Because the opportunistic fungus *C. albicans* is a member of normal flora, it is thought that *C. albicans* growth is held in control by other microorganisms as well as the hosts’ immune system. Patients that are immunocompromised or those being treated with broad spectrum antibiotics can create an environment allowing *C. albicans* the opportunity to expand its growth due to the reduction of factors secreted by other organisms that would normally suppress filamentous growth. In order to understand how both Gram positive and Gram negative bacteria impair *C. albicans* ability to filament we performed a genetic screen using haploinsufficiency to identify common targets in *C. albicans* that are used by both types of bacteria to impair *C. albicans* growth. Though it is an imperfect means to screen for all possible genes related to a particular phenotype due to the diploid nature of *C. albicans*, haploinsufficiency has been used previously to identify strains showing reduced or increased filamentation [34] as well as changes in fitness related to drug susceptibility and growth rates [44]. Using this approach we were successful in identifying 107 genes/ORFs that likely play some role in *C. albicans* filamentation response in the presence of various bacterial species.

The genes identified from this screen fell into a variety of biological functions including adhesion, cell cycle, enzymatic activity, signaling, transcription, and transport. For example, the identification of several genes involved in various types of transport was quite surprising. Several of the genes we identified were associated with the plasma membrane (*CDR4, RTA3*, and *GNP3*) while others were associated with nuclear (*CRM1, POM152*) or mitochondrial (*MGE1, SSU1*, orf19.7358) transport. The transporters associated with the plasma membrane likely are used by *C. albicans* for import/export of bacterial metabolites and QS molecules from the surrounding environment. We speculate that mutations in the *CRM1* or *POM152* genes may be interfering with export/import of signaling proteins or transcriptional regulators required to respond to the presence of bacteria in the environment. It is quite possible that *MGE1, SSU1*, or orf19.7358 transporters may be involved in transporting bacterial molecules that interfere with several other genes associated with mitochondrial function (*DNM1, GCU1, MRP2*, orf19.346, orf19.1201, orf19.4176, orf194018, orf19.4472, and orf19.7152), as mutants of these genes were also shown to be impaired in the filamentation response to all three bacteria. The *cdr4, rta3* and *gnp3* mutants are interesting in that Rta3p is a predicted flippase, which may participate in lipid molecule translocation across the membrane. Likewise the Gnp3p is predicted to be a high affinity glutamine transporter which could serve as a point for small peptide transport. Additionally, Cdr4p has no characterized functional role even though it is a member of a family of ABC transporters, Cdr1-3p [45,46]. Cdr1p and Cdr2p are important in *C. albicans* drug resistance [46,47], while Cdr3p is involved in import of phospholipids [45]. It is possible that Cdr4p plays a role in the import of small peptides or other molecules as ABC transporters have been implicated in peptide transport in *S. aureus* as well as autoinducer-2 transport in *E. coli* [48,49]. We did observe that heterozygous and homozygous mutants of *CDR4* display identical phenotypes to that of our original library isolate, further implicating its role in *C. albicans* interaction with bacteria. Surprisingly, *CDR4* expression was only induced when *S. aureus* was present in the environment with *C. albicans*. *S. aureus*, along with several other Gram positive bacteria, is known to produce small peptide QS molecules. It may be that close contact with *C. albicans* results in the liberation of these molecules from *S. aureus* and *C. albicans* increases its expression of *CDR4* in response to these peptides. This may also be specific to Gram positive bacteria, for example, when *C. albicans* is co-cultured with *Lactobacillus rhamnosus* or *Lactobacillus reuteri* there is a two to three fold increase in *CDR4* expression [50], Since we did not observe any major change in *CDR4* expression when *C. albicans* was grown with *E. coli* or *P. aeruginosa*, may indicate that these organisms don’t produce a peptide required for *CDR4* induction. But the fact that mutants lacking *CDR4* or have reduced expression of the protein may indicate that Cdr4p is playing a role as an importer of bacterial peptides and other molecules as well as *C. albicans* inability to block filamentation in response to the presence of bacteria is a direct result of Cdr4p’s absence.

A second categorical group identified from the screen is the four genes (*ALS6, EAP1, PGA28*, and orf19.5813) that are associated with adhesion. Previous work had found that the attachment of *C. albicans* to *S. gordonii* involves multiple interactions between several components of the bacterial cell wall and *C. albicans* components [51]. Recently, it had been shown the *EAP1* and *ALS3* genes, when expressed in *S. cerevisiae*, conferred the ability of *S. gordonii* to attach to *S. cerevisiae* [52]. The *ALS3* gene has also been shown to be involved in mediating aggregation and, more specifically, directing the attachment of *S. aureus* to the hyphal form of *C. albicans* [53]. The identification of the *eap1* mutant in our screen may indicate that the protein plays not only a role in adherence to bacterial surfaces but may somehow be linked to *C. albicans* ability to “sense” other organisms in its environment. *ALS6* belongs to the *ALS* family of adhesins involved in attachment to biotic and abiotic surfaces, biofilm formation, and virulence, however the specific role of *ALS6* is unknown at this time [52–54]. Though we did not identify an *als3* mutant, the identification of only *als6* in our screen may also signify some importance with regards to attachment and “sensing” of bacteria in the environment. It is possible that other members of the *ALS* gene family could play some role in attachment and “sensing” of bacteria in the environment but due to the nature of this screen, the reduction in gene expression of one copy of other *ALS* genes may be insufficient to create a haploinsufficient phenotype. To our knowledge, no implication of the *ALS* gene family in signal transduction has been suggested in the literature. The ability of these proteins to “sense” bacteria would also add a new functional role for these adhesion proteins with a relation to signal transduction. As with *CDR4* we did confirm our original screen results with heterozygous and homozygous null mutants of *ALS6* further lending credibility to its involvement in *Candida* -bacterial interactions. However unlike *CDR4* we did not see any induction of *ALS6* in the presence of the bacteria. Regardless, it is clear that *ALS6* plays some role in either the adherence to bacteria or “sensing” bacteria in the environment and is somehow able to convey that response by inhibiting *Candida* filamentation.

We were quite surprised to identify multiple insertions within the RPS and RB2 regions of the MRS. In the original use of the haploinsufficiency screen by Uhl et al [34], they also identified insertions within these regions from four clones that either increased or decreased filamentation under their experimental conditions. They identified one ORF they termed *FGR6* (fungal growth regulator) within the RB2 region, though as to its actual function nothing is currently known. There are eight copies of this gene in the genome that all reside within the RB2 region of the MRS elements [37,55]. It has been suggested that mutations affecting the *FGR6* family of genes phenotypically are not believed to be due to haploinsufficiency but some type of dominant regulatory mechanism [55]. Though we have no evidence, it is also possible that insertions of the Tn7 elements into the MRS regions could locally destabilize a region of a chromosome, inadvertently causing a chromosomal translocation/rearrangement thereby affecting the observed phenotype in this screen.

The identification of 18 non-coding/unannotated transcripts also was unexpected. These transcripts were previously identified using RNA-seq and gene tiling studies on the *C. albicans* genome [39,40]. These non-coding or unannotated transcripts may represent small protein coding genes with fewer than 90 amino acids or could possibly be new types of regulatory RNAs that may represent an additional level of control on the morphogenic process. Currently, little to nothing is known about the role of non-coding transcripts or small ORFs below 90 amino acids and their role in *C. albicans* morphogenesis. However, the idea that several of these transcripts could code for regulatory RNAs is intriguing. None of the transcripts appear to be transcribed as antisense with other known ORFs so it is possible that they could play a role in RNA silencing. Nevertheless, there has been some controversy about whether RNA silencing actually occurs in *C. albicans*. Initially, using cell extracts, it had been shown that *C. albicans* does have a Dicer-like activity [56]. A second study demonstrated that *in vivo* production of a dsRNA hairpin to interfere with the *ADE2* gene resulted in no observable gene silencing [57]. Furthermore, characterization of the *DCR1* dicer gene of *C. albicans* demonstrated that this activity is primarily associated with rRNA and and snRNA processing [58]. So, it appears that if the transcripts we have reported here have any impact on RNA silencing of RNA pol II transcribed genes the mechanism is likely unknown or does not exist in *C. albicans*.

This study has allowed us to develop the framework for future delineation of the genetic and signaling events that occurs between *Candida* and bacteria. We postulate that this genetic overlap with response to different bacterial species involves several common pathways that *C. albicans* utilizes for communication. However, several questions remain. For example, do the mutants we identified show a lack of response to bacteria in their environment due to a single secreted bacterial metabolite or QS molecule, or is it a combination of several molecules that exert the inhibitory action on *C. albicans*? It is well documented that single molecules from bacteria can inhibit *C. albicans* filamentation under laboratory conditions [11,12,14,16,17], but it is not well understood if these molecules are present in the environment at all times, which could indicate a combination of effectors may be required in the environment. Also, do the mutants that we have identified in this study also play a role in *C. albicans* QS regulation of filamentation or is that a separate phenomenon? We believe that it is likely that *C. albicans* uses some of these genes to regulate its own QS response as it doesn’t make sense that *C. albicans* would have developed separate molecular machinery to respond to both types of stimuli. We hope to address these questions in future studies. We believe that in understanding the mechanisms of action of both bacterial QSMs and other metabolites on *C. albicans* biology may lead to development of novel ways to control *C. albicans* growth in critically ill patients in addition to current antifungal therapies.

## Supporting Information

Table S1 Genetic elements identified from the *C. albicans* Tn7 insertion library screen.
(DOCX)

Table S2 Major repeat sequences identified from the *C. albicans* Tn7 library screen.
(DOCX)

Table S3 Non-coding/unannotated transcripts identified in *C. albicans* Tn7 library screen.
(DOCX)
