# Supplementary material for: Characterization of Genetic Determinants That Modulate Candida albicans Filamentation in the Presence of Bacteria
Source: PLoS One. 2013 Aug 7;8(8):e71939. doi: 10.1371/journal.pone.0071939 (PMC3737206; doi:10.1371/journal.pone.0071939)
Supplement: Table S1 — Genetic elements identified from the C. albicans Tn7 insertion library screen. (DOCX) [file pone.0071939.s001.docx]

| **Table S1.** Genetic elements identified from the C. albicans Tn7 insertion library screen | | | | | |
| --- | --- | --- | --- | --- | --- |
| **Systematic** | **Standard** | **Tn7** | **Library** | **Predicted/Known** | **Predicted/Known** |
| **Name** | **Name** | **Location** | **Number** | **Biological Function** | **Protein Localization** |
| orf19.10 | *ALK8* | ORF | 166a9 | Metabolic processes | Endoplasmic Reticulum |
| orf19.23 | *RTA3* | 5’ of ORF | 4g8 | Response to stress | Membrane |
| orf19.344 | *-------* | 3’ of ORF | 181a2 | Unknown | Unknown |
| orf19.346 | *-------* | 3’ of ORF | 158c12 | Metabolic processes | Cytoplasm |
| orf19.470* | *-------* | ORF | 133f7 | Transcription | DNA binding complex |
| orf19.511 | *-------* | 5’ of ORF | 125c6 | Metabolic processes | Nucleus |
| orf19.517 | *HAP31* | 5’ of ORF | 159f4 | Transcription | DNA binding complex |
| orf19.526 | *NHP2* | ORF | 128d3 | RNA processing | RNA binding complex |
| orf19.536 | *-------* | 3’ of ORF | 122e7 | Transcription | DNA binding complex |
| orf19.860 | *BMT8* | 3’ of ORF | 129a8 | Filamentous growth | Unknown |
| orf19.978 | *BDF1* | 5’ of ORF | 177c11 | Transcription | Chromosome |
| orf19.999 | *GCA2* | ORF | 133d12 | Metabolic processes | Endoplasmic Reticulum |
| orf19.1040 | *MAD2* | ORF | 125h1 | Cell cycle | Chromosome |
| orf19.1110 | *-------* | 5’ of ORF | 138d11 | Metabolic processes | Cytoplasm |
| orf19.1201 | *-------* | 5’ of ORF | 184e2 | RNA processing | Mitochondria |
| orf19.1368 | *-------* | 3’ of ORF | 75b1 | Unknown | Unknown |
| orf19.1401 | *-------* | 5’ of ORF | 42f9 | Adhesion | Membrane |
| orf19.1523 | *FMO1* | ORF | 180c12 | Drug response | Unknown |
| orf19.1693 | *CAS4* | ORF | 154d11 | Signaling | Cell tip |
| orf19.1728 | *-------* | ORF | 162f11 | Unknown | Unknown |
| orf19.1759 | *PHO23* | ORF | 134b7 | Transcription | RNA binding complex |
| orf19.1911 | *PGA52* | ORF | 134c6 | Unknown | Membrane |
| orf19.1941 | *NUF2* | 5’ of ORF | 135b3 | Cell cycle | Chromosome |
| orf19.1996* | *CHA1* | ORF | 70a7 | Filamentous growth | Unknown |
| orf19.2038 | *-------* | 3’ of ORF | 178a8 | Unknown | Unknown |
| orf19.2081 | *POM152* | ORF | 75d8 | Transport | Membrane |
| orf19.2106 | *-------* | ORF | 160b6 | Unknown | Unknown |
| orf19.2114 | *-------* | ORF | 134g2 | Metabolic processes | Nucleus |
| orf19.2138 | *ILS1* | ORF | 49c11 | RNA processing | Cytosol |
| orf19.2290 | *TOR1* | ORF | 98f7 | Signaling | TOR complex |
| orf19.2356 | *CRZ2* | ORF | 74e11 | Transcription | Intracellular |
| orf19.2417* | *SMC5* | ORF | 61e7 | DNA processing | Nucleus |
| orf19.2423 | *ZCF11* | ORF | 149b7 | Transcription | Nucleus |
| orf19.2495 | *GSL1* | ORF | 93h4 | Metabolic processes | Membrane |
| orf19.2524 | *MGE1* | 5’ of ORF | 156f2 | Protein processing | Mitochondria |
| orf19.2579 | *-------* | ORF | 184c4 | DNA processing | Chromosome |
| orf19.2929 | *GSC1* | 3’ of ORF | 156b8 | Metabolic processes | Membrane |
| orf19.2982 | *-------* | ORF | 69g10 | Autophagy | Unknown |
| orf19.2989 | *GOR1* | 3’ of ORF | 96g2 | Metabolic processes | Nucleus |
| orf19.3001 | *TEM1* | ORF | 134c1 | Cell cycle | Spindle pole body |
| orf19.3087.2 | *-------* | ORF | 161e5 | Unknown | Unknown |
| orf19.3100 | *-------* | 3’ of ORF | 156f1 | Unknown | Unknown |
| orf19.3124 | *-------* | ORF | 131a2 | Protein processing | Ribosome |
| orf19.3394 | *-------* | ORF | 134d10 | Unknown | Membrane |
| orf19.3519 | *SUA72* | ORF | 145c6 | Transcription | Unknown |
| orf19.3643 | *-------* | ORF | 131b1 | Unknown | Intracellular |
| orf19.3730 | *-------* | ORF | 120c7 | Protein processing | Endoplasmic Reticulum |
| orf19.3767 | *-------* | ORF | 158c10 | Protein processing | Golgi |
| orf19.3791 | *FGR10* | ORF | 143h2 | Filamentous growth | Cytosol |
| orf19.4018* | *-------* | ORF | 116b7 | Translation | Ribosome |
| orf19.4023 | *MRP2* | ORF | 143d4 | Translation | Ribosome |
| orf19.4054 | *CTA24* | ORF | 125c9 | Transcription | Unknown |
| orf19.4086 | *-------* | 3’ of ORF | 126g4 | Unknown | Cytoplasm |
| orf19.4099 | *ECM17* | ORF | 95b6 | Metabolic processes | Cytosol |
| orf19.4112 | *-------* | 3’ of ORF | 135c1 | Metabolic processes | Cytosol |
| orf19.4119 | *SPO72* | ORF | 175e7 | Autophagy | Membrane |
| orf19.4176 | *-------* | ORF | 147h9 | Translation | Ribosome |
| orf19.4246 | *-------* | ORF | 68d3 | Metabolic processes | Unknown |
| orf19.4261 | *TIF5* | 5’ of ORF | 127f6 | Translation | Ribosome |
| orf19.4263 | *-------* | ORF | 173g2 | Unknown | Unknown |
| orf19.4412 | *-------* | ORF | 120d11 | DNA processing | Chromatin |
| orf19.4610 | *-------* | ORF | 161g5 | Proteolysis | Unknown |
| orf19.5076 | *CDR4* | 5’ of ORF | 35d10 | Transport | Membrane |
| orf19.5101* | *CCR4* | 5’ of ORF | 133d6 | DNA processing | DNA binding complex |
| orf19.5144 | *PGA28* | 5’ of ORF | 97h8 | Unknown | Cell surface |
| orf19.5169* | *-------* | 5’ of ORF | 94b8 | Unknown | Nucleus |
| orf19.5212 | *-------* | ORF | 81c7 | Unknown | Unknown |
| orf19.5407* | *SOF1* | ORF | 68e6 | RNA processing | Ribosome |
| orf19.5485 | *-------* | ORF | 134f5 | DNA processing | DNA binding complex |
| orf19.5506 | *PLC1* | ORF | 134g3 | Signaling | Cytoplasm |
| orf19.5519 | *GCV1* | 5’ of ORF | 162d1 | Metabolic processes | Mitochondria |
| orf19.5665 | *-------* | ORF | 134d9 | Metabolic processes | Unknown |
| orf19.5799 | *-------* | ORF | 170c3 | Unknown | Unknown |
| orf19.5813 | *-------* | 3’ of ORF | 125h3 | Unknown | Unknown |
| orf19.5897 | *-------* | 5’ of ORF | 168h5 | Unknown | Cytosol |
| orf19.5902* | *RAS2* | ORF | 109b6 | Signaling | Membrane |
| orf19.5938 | *SEN1* | ORF | 134b8 | RNA processing | RNA binding complex |
| orf19.6082 | *-------* | ORF | 175a5 | Protein processing | Endoplasmic Reticulum |
| orf19.6323 | *HPA2* | 5’ of ORF | 88a4 | Metabolic processes | Nucleus |
| orf19.6488* | *-------* | ORF | 72d12 | Unknown | Unknown |
| orf19.6592 | *-------* | 3’ of ORF | 176f2 | Transport | Membrane |
| orf19.6722* | *-------* | ORF | 78e8 | DNA processing | Cytosol |
| orf19.6736 | *-------* | 5’ of ORF | 78g8 | Unknown | Mitochondria |
| orf19.6747 | *-------* | ORF | 175c8 | Transport | Cytoplasm |
| orf19.6785 | *RPS12* | ORF | 189h2 | Translation | Ribosome |
| orf19.6931 | *-------* | ORF | 70d10 | RNA processing | RNA binding complex |
| orf19.6968* | *-------* | ORF | 78g11 | Unknown | Unknown |
| orf19.6987 | *DNM1* | ORF | 76a4 | Mitochondrial | Mitochondria |
| orf19.7085 | *-------* | 3’ of ORF | 176h6 | Unknown | Unknown |
| orf19.7108 | *RPS18* | 3’ of ORF | 187a10 | Translation | Ribosome |
| orf19.7130 | *-------* | ORF | 125h4 | Unknown | Unknown |
| orf19.7152 | *-------* | 5’ of ORF | 185g12 | Metabolic processes | Mitochondria |
| orf19.7232 | *IRR1* | 3’ of ORF | 94h8 | Cell cycle | Nucleus |
| orf19.7301 | *-------* | ORF | 134d4 | DNA processing | Unknown |
| orf19.7313 | *SSU1* | 5’ of ORF | 168e11 | Transport | Membrane |
| orf19.7342 | *AXL1* | ORF | 169d5 | Proteolysis | Unknown |
| orf19.7358 | *-------* | 3’ of ORF | 98d10 | Protein processing | Mitochondria |
| orf19.7413 | *MMS21* | 5’ of ORF | 176a10 | DNA processing | DNA binding complex |
| orf19.7414* | *ALS6* | 5’ of ORF | 49b8 | Adhesion | Cell surface |
| orf19.7483 | *CRM1* | ORF | 149a8 | Transport | Nucleus |
| orf19.7512 | *-------* | ORF | 131a3 | Metabolic processes | Unknown |
| orf19.7565 | *GNP3* | ORF | 99e9 | Transport | Membrane |
| orf19.7567 | *-------* | ORF | 156e4 | Unknown | Unknown |
| orf19.7572* | *SPT7* | ORF | 42h5 | Protein processing | Mitochondria |
| orf19.7579 | *FGR34* | ORF | 151a9 | Filamentous growth | Unknown |
| orf19.7657 | *POP3* | ORF | 90c8 | RNA processing | RNA binding complex |
| orf19.7667* | *IAH1* | 5’ of ORF | 131c7 | Metabolic processes | Cytoplasm |

*denotes gene was identified in multiple candidates. Library number denotes the plate number and well number location of the candidate strain so that, if requested, individual candidates can be located.
