# Supplementary material for: Characterization of Genetic Determinants That Modulate Candida albicans Filamentation in the Presence of Bacteria
Source: PLoS One. 2013 Aug 7;8(8):e71939. doi: 10.1371/journal.pone.0071939 (PMC3737206; doi:10.1371/journal.pone.0071939)
Supplement: Table S2 — Major repeat sequences identified from the C. albicans Tn7 library screen. (DOCX) [file pone.0071939.s002.docx]

| **Table S2.** Major repeat sequences identified from the *C. albicans* Tn7 library screen | |
| --- | --- |
| **Major repeat sequence** | **Library number** |
| MRS-R | 71a3, 72f5 |
| MRS-1 | 71c7, 76a5, 134c9, 134h11, 161a10 |
| MRS-2 | 56a9, 70a3, 81a4, 81f9, 120h2, 124g9, 129f3, 132c7, 133b6, 134a7, 134b3, |
|  | 134c10, 136f9, 142e7, 143d10, 151b11, 163a4, 165d11 |
| MRS-4 | 113f8, 119g3, 123e10, 133g9, 134e9, 144g9, 165e11 |
| MRS-6 | 133c5 |
| MRS-7a | 42f11, 124d4, 124g12, 138d12 |
| MRS-7b | 71b11, 176g8 |
