# Supplementary material for: Characterization of Genetic Determinants That Modulate Candida albicans Filamentation in the Presence of Bacteria
Source: PLoS One. 2013 Aug 7;8(8):e71939. doi: 10.1371/journal.pone.0071939 (PMC3737206; doi:10.1371/journal.pone.0071939)
Supplement: Table S3 — Non-coding/unannotated transcripts identified in C. albicans Tn7 library screen. (DOCX) [file pone.0071939.s003.docx]

| **Table S3.** Non-coding/unannotated transcripts identified in *C. albicans* Tn7 library screen | | |
| --- | --- | --- |
| **Transcript name** | **Reference** | **Library number** |
| Novel-Ca21chrR-009 | Bruno et al. | 145c8 |
| Novel-Ca21chrR-023 | Bruno et al. | 134b12 |
| Novel-Ca21chrR-093* | Bruno et al. | 12e3 |
| Novel-Ca21chrR-094 | Bruno et al. | 75e5 |
| Novel-Ca21chrR-100 | Bruno et al. | 182c1 |
| Novel-Ca21chr2-037 | Bruno et al. | 134g1 |
| Novel-Ca21chr7-001 | Bruno et al. | 148b5 |
| TFRW255 | Sellam et al. | 135b6 |
| TF1W4 | Sellam et al. | 159e10 |
| TF1W160 | Sellam et al. | 98b5 |
| TF3C2 | Sellam et al. | 61e3 |
| TF3C162 | Sellam et al. | 183h1 |
| TF4C158* | Sellam et al. | 134f6 |
| TF5C101 | Sellam et al. | 76f10 |
| TF5W116 | Sellam et al. | 134c3 |
| TF5W130 | Sellam et al. | 166e9 |
| TF7C126 | Sellam et al. | 95h6 |
| TF7W6 | Sellam et al. | 88d10 |

*denotes transcript was identified in multiple candidates
